# Colorectal Adenomas—Genetics and Searching for New Molecular Screening Biomarkers

**DOI:** 10.3390/ijms21093260

**Published:** 2020-05-05

**Authors:** Anna Siskova, Klara Cervena, Jan Kral, Tomas Hucl, Pavel Vodicka, Veronika Vymetalkova

**Affiliations:** 1 Department of Molecular Biology of Cancer, Institute of Experimental Medicine, Videnska 1083, 14200 Prague, Czech Republic; klara.cervena@iem.cas.cz (K.C.); jan.kral@ikem.cz (J.K.); veronika.vymetalkova@iem.cas.cz (V.V.); 2 Institute of Biology and Medical Genetics, First Faculty of Medicine, Charles University, Albertov 4, 12800 Prague, Czech Republic; 3 Institute for Clinical and Experimental Medicine, Videnska 1958/9, 14021 Prague, Czech Republic; tomas.hucl@ikem.cz; 4 Biomedical Centre, Faculty of Medicine in Pilsen, Charles University, Alej Svobody 76, 32300 Pilsen, Czech Republic

**Keywords:** colorectal adenoma, colorectal cancer, biomarkers, early detection

## Abstract

Colorectal cancer (CRC) is a malignant disease with an incidence of over 1.8 million new cases per year worldwide. CRC outcome is closely related to the respective stage of CRC and is more favorable at less advanced stages. Detection of early colorectal adenomas is the key to survival. In spite of implemented screening programs showing efficiency in the detection of early precancerous lesions and CRC in asymptomatic patients, a significant number of patients are still diagnosed in advanced stages. Research on CRC accomplished during the last decade has improved our understanding of the etiology and development of colorectal adenomas and revealed weaknesses in the general approach to their detection and elimination. Recent studies seek to find a reliable non-invasive biomarker detectable even in the blood. New candidate biomarkers could be selected on the basis of so-called liquid biopsy, such as long non-coding RNA, microRNA, circulating cell-free DNA, circulating tumor cells, and inflammatory factors released from the adenoma into circulation. In this work, we focused on both genetic and epigenetic changes associated with the development of colorectal adenomas into colorectal carcinoma and we also discuss new possible biomarkers that are detectable even in adenomas prior to cancer development.

## 1. Introduction

Colorectal cancer (CRC) is a serious heterogeneous disease that stands in third place in cancer incidence and represents the second cause of death in the world (nearly1.8 million patients newly diagnosed and 1 million patients who die every year) [1]. CRC has become predominant cancer in Western countries, which could be partially explained by the aged population and adverse lifestyle habits such as smoking, increased consumption of red meat and alcohol, lack of physical activity related to obesity, and diabetes, connected usually with low diversity of intestine microflora. Risk factors also include positive family history reflecting individual genetic equipment [2]. The screening programs aim to identify patients with precancerous lesions or those with resectable CRC stages 0, I, and II, who have a generally better prognosis than symptomatic patients with the pre-existing disease [3]. Despite screening programs, many patients are diagnosed in the stages III and IV of CRC that lead to a worse overall prognosis [4]. According to data from National Cancer Institute from the United States of America (USA) [5], five years survival rates for stage IV account only for 12% at colon cancer (CC) and 13% at rectal cancer (RC), while detection at an early stage I can increase the chance to survive up to 92% at CC and 88% at RC [6].

Several screening methods such as stool testing, blood testing, and endoscopic and radiological examination are currently available [7,8]. Polyps or tumors can manifest by microscopic bleeding (so-called occult bleeding). First-line test detecting occult bleeding is a fecal occult blood test (FOBT). Guaiac fecal occult blood test (gFOBT) detects hemoglobin (Hb) by peroxidase activity. Nevertheless, gFOBT is insufficiently specific to human hemoglobin and connected with a risk of false-positive results and omission of small polyps or non-bleeding polyps [7]. Despite its disadvantages, it has been able to contribute to a 33% reduction in CRC mortality [9]. Nowadays, gFOBT has predominantly been replaced by a fecal immunochemical test (FIT) based on antibody assay, which provides qualitative and quantitate results on Hb concentration per gram feces. Positive results of gFOBT or FIT are followed by endoscopic examination [8].

Endoscopic methods include colonoscopy examination, sigmoidoscopy, or capsule endoscopy. Colonoscopy is the main investigative method of the large bowel, this technique provides visualization of the entire large intestine, precise localization, biopsy, or complete removal of a potential precancerous lesion in a single session [10]. Early polypectomy leads to a 76–90% reduction in CRC incidence [11]. The weakness of this method is its invasiveness, it is an unpleasant procedure requiring several days of diet restriction and bowel preparation. These could pose an obstacle for many people, and among other things, it is expensive with the necessary presence of a very well-trained examiner [12].

Sigmoidoscopy compared to colonoscopy reduces time-consuming examination and patient discomfort and provides a lower risk of complications without the need for sedation, but allows investigation of only the rectum and the sigmoid. The study of sigmoidoscopy screening of individuals between 55 and 64 years in the United Kingdom (UK), indicated subsequent CRC incidence reduction by 33% and mortality by 43% [13].

Colon capsule endoscopy (CCE) is a non-invasive method suitable for individuals who are unwilling to undergo colonoscopy because of discomfort or any other obstacles. Meta-analysis showed that CCE for any polyp has a specificity of 89% and sensitivity of 73%. Though CCE is not as accurate as colonoscopy, it could decrease the need for its application [14].

CRC screening by radiology using computed tomographic (CT) colonography is able to visualize the entire colorectum and with no need for sedation. Even though it still requires bowel preparation, it is a relatively non-invasive method. This technique can detect only large adenomas and tumors with size ≥10 mm, nevertheless with sensitivity of 90% [15].

Screening methods based on blood testing were enriched by a highly promising biomarker, methylated gene *septin9* (^m^SEPT9) in the last few years. ^m^SEPT9 is released from CRC cells into circulation and is detectable in peripheral blood. A recent study showed that ^m^SEPT9 assay, approved by Food and Drug Administration (FDA) in the USA, has a higher specificity (94.5%) than FOBT at advanced stages of CRC, but not at asymptomatic patients with early neoplasia [16,17].

Several types of a lesion can be histologically described from the colonoscopy biopsy. A colon polyp is a small clump of cells that forms on the lining of the colon epithelium. There are two main classes of polyps, non-neoplastic and neoplastic (Table 1) [18]. In general, the larger the neoplastic adenoma the greater the risk of cancer. Table 2 shows the recommended follow up after patient polypectomy [19,20,21]. Although the recommended surveillance guideline has been widely accepted, clinicians still detect the incidence of CRC (<10%) developed during the initial colonoscopy and the subsequent follow-up examination. This subgroup of CRCs is referred to as interval CRC (I-CRC) and represents one of the problems that screening programs face [22].

Around 5–10% of CRC cases are related to heredity including most common syndromes such as hereditary non-polyposis colorectal cancer (HNPCC), familial adenomatous polyposis (FAP) and attenuated familial adenomatous polyposis (aFAP), MUTYH-associated polyposis (MAP), Juvenile polyposis syndrome (JPS), Peutz-Jeghers syndrome (PJS), Polymerase proofreading-associated polyposis (PPAP), PTEN hamartoma tumors syndrome (PHTS), Cowden syndrome, and Familial colorectal cancer type X, while more than 90% of CRC cases are of sporadic origin [6,7]. Syndromes are usually detected at an early age. However, sporadic CRC correlates with increasing age due to the accumulation of mutations in intestine cells [23,24]. 

**Figure 1:**
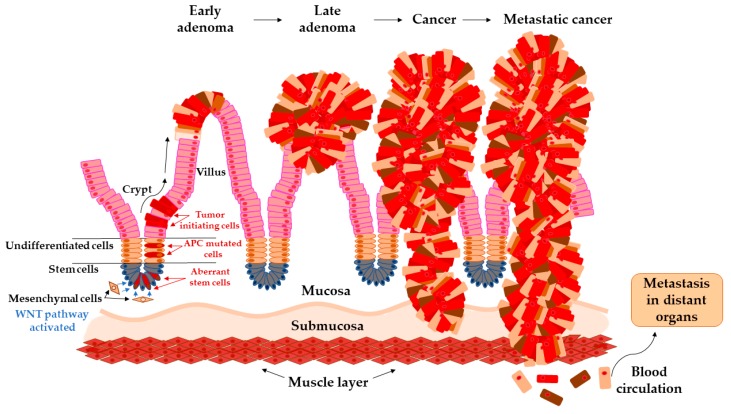
Arise of tumor-initiating cells from aberrant colon crypt and subsequent transition of early adenoma to metastatic cancer.

In the study by Brenner et al. [25], 10 years of cumulative risk of CRC among both sex with advanced adenomas increases from 25.4–25.2% at age 55 years to 42.9–39.7% at age 80 years. The development of carcinoma from adenoma tissue can last 5 to 20 years, and it is not influenced purely by one pathway [26,27]. This transition is a complex, multifactorial process that has been characterized by chromosomal instability (CIN), microsatellite instability (MSI), and DNA methylation in CpG islands areas (CIMP). All these pathways may overlap with each other and are responsible for genetic instability in adenoma that could undergo malignant transformation [28] (Figure 1). The events contributing to these processes are constantly subject to intensive investigations [27].

Considering the current knowledge about the CRC development and with an application of screening programs, we are still missing identification of patients with asymptomatic disease progression in early stages, where detection plays a key role in cancer survival. Recent studies seek to find new non-invasive biomarkers measurable even in early stages of CRC from an area of non-coding RNA, inflammatory biomarkers, or cell-free DNA [29].

### Transition of Adenoma to Carcinoma in Colon

The colon epithelium is constantly and rapidly renewing tissue. Old cells on the top of the villus are released into the lumen and replaced with new cells raised from colonic crypts. On the bottom of colonic crypts are stem cells that proliferate and differentiate into the cellular compartment of colon epithelium [31]. Vogelstein et al. [32] proposed the classical model of tumor evolution in the large bowel (Figure 1). Cells with high WNT signaling activity arise from aberrant crypts and evolve into a tubular or tubule-villous polyp. The subsequent proliferation of polyp may lead to the development of early adenoma with a low grade of dysplasia. Early adenoma expanses into advanced adenoma with a high grade of dysplasia and with increasing accumulation of mutations in daughter cells progressing ultimately further into carcinoma [2,32,33]. 

Each mutation that provides tumor cell-selective growth advantage is called driver mutation. This advantage slightly increases the growth rate of clonal expansion around 0.4% and is increasing with every new driving mutation [34]. Driver mutations enhance the accumulation of a large number of somatic mutations due to altering the cell condition and reduce the population fitness landscape. The predominant mutations, so-called passenger mutations, are mutations without selective growth advantage. With each clonal expansion of cancer cells, heterogeneous passenger mutations are generated that constitute the enormous variations of unique tumors [35].

Thanks to the next-generation sequencing (NGS) technique, thousands of mutations in the human genome were identified and some of them contribute to malignant evolution [36]. The driver mutations in the *APC* gene, predominantly frameshift at codon 1,554 [37], provide cell-selective growth advantage [32], and cause loss of cell ability to control the concentration level of protein β-catenin in the cytoplasm. β-catenin implements in the WNT signaling pathway and its concentration imbalances lead to uncontrolled growth and cell division [38]. Following mutations in *TP53* or *SMAD4* genes induce transformation into a malignant tumor, which overgrows into basal tissue and has an ability to metastasize into lymph nodes and distant organs [27].

## 2. Genetic Changes in Adenoma

The evolution of adenoma to carcinoma contains a wide range of genetic and epigenetic alterations. Here, we described the most relevant genetic changes associated with precancerous stages of colorectal adenoma.

### 2.1. Chromosomal Instability (CIN)

Chromosomal instability is associated with about 70% of sporadic CRC cases and is caused by aberrant segregation during mitoses, breaks in DNA due to nucleotide excision repair genes (NER) deficiency, or fusion of telomeres.

Chromosomal rearrangement could be classified as a numerical CIN, involving gains or losses of whole chromosomes, or it could be described as a structural CIN involving translocations, inversions, amplifications, or deletions certain parts of chromosomes [39]. CIN acts as a cancer driver by changing the copy number of large gene cohorts within tumor suppressor genes, oncogenes, DNA repair genes, and apoptotic genes [40]. Besides, the loss of one of the parental alleles during mitosis has a consequence in the loss of heterozygosity (LOH) [41].

In the study by Hermsen et al. chromosomal aberrations of 66 non-progressed colorectal adenomas, 46 progressed adenomas, and 36 colorectal carcinomas were analyzed by comparative genomic hybridization (CGH) method [42]. Authors observed that even in small adenomas a certain degree of CIN was found, independent of the degree of dysplasia. In particular, losses of chromosomal regions were observed in small non-progressed adenomas while in progressed adenomas predominantly gains of chromosomal regions and increased CIN were detected. The higher accumulation of losses at 8p21-pter, 15q11-q21, 17p12-13,18q12-21 and gains at 8q23-qter, 13q14-3, 20q13 chromosomes correlated with tumor progression [42]. Further, the most common losses were found at 1p, 4, 8p, 14, 15, 17p, 18, and most common gains at chromosomes 7, 8q, 13, 20 [43].

### 2.2. Microsatellite Instability (MSI)

MSI is defined as the change in microsatellite length, caused by the insertion or deletion of repetitive sequences in a tumor compared to the length of microsatellite in non-malignant tissue in the same individual. MSI is caused by a deficiency of the DNA repair mechanism, particularly the mismatch repair pathway (MMR). Under normal physiological conditions, the role of MMR is to correct DNA errors formed during the replication process, however deactivating some MMR genes (e.g., *MLH1, MSH2, MSH6,* or *PMS1,* and *PMS2*) results in MSI [44]. 

Originally, MSI has been reported to be associated with germline mutation of MMR genes in hereditary non-polyposis colorectal syndromes (HNPCC) known as a Lynch syndrome (LS). Sporadic tumors with MSI, commonly caused by biallelic promoter hypermethylation of the *MHL1* gene, has a tendency to arise in the proximal colon. The frequency of the MSI is 80% to 95% in HNPCC cancers and 10% to 15% in sporadic CRC [45,46].

HNPCC tumors demonstrate high MSI, especially those located in the proximal colon, and the presence of tumor-infiltrating lymphocytes, nevertheless associated with better prognosis [47].

### 2.3. DNA Methylation in CpG Islands

DNA methylation, an important epigenetic modification, is closely related to the occurrence and development of tumors [48] and takes place at the 5-position of the pyrimidine ring of the cytosine residues within CpG sites to form 5-methylcytosines. CpG islands, 0.5- to 2-kb regions rich in cytosine-guanine dinucleotides, are present in approximately half of all human genes; comprising about 30,000 CpG islands in the human genome. The presence of multiple methylated CpG sites in CpG islands leads to the stable silencing of gene expression [49,50,51]. The CpG island methylator phenotype (CIMP) was firstly introduced in CRC by Toyota et al. [52] as a mechanism of CRC development. CIMP-positive CRC is characterized by a high degree of methylation in multiple CpG islands of genes associated with CRC, such as tumor suppressor genes *MLH1*, *MGMT*, and *p16* [52,53].

CIMP level corresponds to the histological stage of dysplasia. In the study of Rashid [54], the methylation status of *p16*, MINT2, and MINT31 was determined and the methylation of these loci was present at 41% tubular adenomas and at 73% of tubulo-villous or villous adenomas. Interestingly, tubulo-villous and villous types of adenomas were more frequently found to evolve into invasive carcinomas. *K-ras* mutation was also observed in larger adenomas associated with higher CIMP [53,54], and this mutation also occurred at sessile serrated adenomas with significantly frequent methylation of MINT1 and MINT2 genes [55,56].

Although it is well known that hypermethylation of *MHL1* gene leads to MSI, these two pathways, CIMP and MSI, are shown to be independent ways. It has been suggested that hypermethylation of *MHL1* gene is a consequence in late stages, which was supported by the presence of previous mutations or allelic loss [54,57]. 

## 3. Insight into Novel Candidate Biomarkers of CRC

Research on CRC reported during the last decade has improved our understanding of the etiology and development of colorectal neoplasia and revealed weaknesses in the general approach to their detection and elimination. 

The aims of recent studies on early CRC detection are oriented on the identification of new biomarkers and a more comprehensive understanding of existing biomarkers. A significant role in this field is played by non-coding RNA (long and small non-coding RNAs), cell-free DNA, circulating tumor cells, and inflammatory agents or length of telomeres. Here, we present the most promising biomarkers, which could serve as a diagnostic tool.

### 3.1. Long Non-Coding RNAs (lncRNAs)

Only 1–2% of the human genome encodes proteins whereas 70–90% is transcribed into non-coding RNA (ncRNA) [58,59]. The role of non-coding RNAs in organisms comprises numerous biological functions such as RNA splicing, regulation of transcription and translation, epigenetic modification, cell metabolism, interaction with RNA, DNA, and proteins [60]. Recently, it has been documented their involvement in a several diseases, especially in cancer. LncRNAs, transcripts longer than 200 nucleotides, can act as oncogenes or tumor suppressor genes in CRC and are involved in all phases of cancer evolution, tumor progression including migration of cancer cells, proliferation, tumor invasion, and metastasis formation [61]. LncRNAs can be detected not only in target tissue but also in peripheral blood. Therefore, these transcripts could represent promising diagnostic biomarkers for CRC and even in precancerous stages. A number of lncRNA e.g., *CCAT1, CAHM, CRNDE, CRCAL1-4, H19, HOTAIR, MALAT1* was found significantly differentially expressed in carcinomas compared to adjacent colon tissue [62,63].

Colon-cancer associated transcript 1 (*CCAT1*) was recently found significantly up-regulated in all stages of adenoma-carcinoma cascades, in adenomatous polyps (100 - fold), in tumors (5 - fold) or in metastases (over - 100 fold) when compared to adjacent mucosa [64]. *CCAT1* locus is located nearby of family *MYC* regulator genes, a well-known transcription factor. Observations of Xiang et al. [65] suggest that *CCAT1-L* lncRNA is involved in *MYC* regulation by intra-chromosome looping between the *MYC* gene promoter and distal upstream enhancer elements that regulate *MYC* transcription. The up-regulation of lncRNA-*CCAT1* is highly abundant in the premalignant stages of CRC [60,66].

The *H19*, an oncofetal gene for lncRNA, is located in an imprinted region of chromosome 11, close to the telomeric region. *H19* is overexpressed during the early stages of embryogenesis, downregulated after birth, and re-expressed during tumor genesis [67]. *H19*-lncRNA regulates gene expression of *CDK4, CCND,* and certain cancer-related proteins, such as RB1, and indirectly the activity of β-catenin via reduction of *CDK8* expression by interacting with macroH2A [68]. H19 lncRNA is highly abundant in many tumors, including CRC [69]. Yoshimizu et al. [70] demonstrated that lack of *H19*-lncRNA expression may be considered as an initiating step in increasing the number of polyp appearance in *APC* mutated carcinogenesis mice model.

A gene locus (Chr16: hCG_1815491), named colorectal neoplasia differentially expressed (*CRNDE*), encodes lncRNA that is activated in early stages in colorectal neoplasia [71]. Elevated expression was detected in more than 90% of colorectal adenomas and adenocarcinomas in comparison to adjacent tissue. Moreover, transcripts of *CRNDE* were also found in the plasma of 13 out of 15 CRC patients [71]. A significant up-regulation of lncRNA, *CRNDE-h* variant transcript was found in serum exosomes of individuals with adenoma and CRC patients compared to control healthy subjects [72]. In addition, the level of *CRNDE*-lncRNA correlated with tumor size and advanced CRC stages and patient survival. *CRNDE* knockout suppressed CRC cell proliferation and supported apoptosis both in vitro and in vivo in the mouse model [73]. *CRNDE*-lncRNA also plays a significant role in CRC development by enhancing an activity of Ras/MAPK and WNT/β-catenin signaling pathways [74].

### 3.2. MicroRNAs (miRNAs)

MicroRNAs (miRNAs), class of small non-coding RNAs (with an average of 22 nucleotides in length), regulate the gene expression through RNA interference. MiRNAs may act as oncogenes or tumor suppressors, and their differential expression has been involved in many cancers, including CRC [75]. Their role in the silencing or triggering several pathways has been observed, thus contributing to the transition from normal epithelial colonic mucosa to adenoma and carcinoma [76]. To date, numerous studies have been focused on the differences in the miRNA’s expression between patients with CRC and healthy individuals. However, the investigation of precancerous adenomas is scarce 2 [77].

Differential miRNAs expression levels between several types of adenomas were described by studies shown in (Table 3).

The study by Tsikitis et al. focused on several types of adenomas characterized by histology and malignant potential. MiR-145, -143, -107a, -194, and -26a exhibited higher expression in low risk adenomas than in high risk adenomas, whereas miR-663b, -1268, -320a, -320b, and -1275 were highly expressed in high risk adenomas. Authors suggested the potential value of comprehensive miRNAs profiling to identify patients with high-risk malignant potential adenomas [78]. Kanth et al. analyzed miRNAs isolated from in formalin-fixed paraffin-embedded (FFPE) samples from 6 patients with sessile serrated polyps (SSA/Ps), hyperplastic polyps (HPs), and paired adjacent colon mucosa by small noncoding RNA sequencing. Several differentially expressed miRNAs (miR-135b, -378a, -548, -31, and -196b) were observed in SSA/Ps in comparison with HPs. The authors suggested that these miRNAs might serve as a good diagnostic biomarker of serrated polyps [79]. Aslam et al. also analyzed miRNAs isolated from FFPE samples and the expression level of miR-135b was progressively increased with the sequential progression of non-affected tissue to adenoma and carcinoma [80]. Oberg et al. identified several miRNAs (including miR-31 and -135) that evinced a significant difference in their expression profiles between adenomas and adjacent mucosa [81]. The involvement of miR-31 in transition from adenoma to carcinoma was also proposed by [82]. 

A study by Wang et al. focused on miRNA as biomarkers for prediction of adenoma recurrence. Patients with advanced colorectal adenomas were monitored for 22–24 months and almost 50% of them experienced adenoma recurrence. Authors identified that low expression of miR-194 can serve as a potential independent factor for adenoma recurrence. Moreover, this parameter was a better predictor than number of adenomas and adenoma size [83].

In the last decade, many studies focused on the concept of liquid biopsy with the goal to identify diagnostic and prognostic biomarkers from body fluids. As miRNAs can be secreted into the circulation, they might represent promising diagnostic candidates [51]. It has been observed that circulating miRNA can also aid to distinguish between CRC and adenoma patients (Table 3). 

Ardila et al. analyzed circulating miRNAs in the serum of advanced adenomas, hyperplastic polyps and controls and found that miR-141, -143, and -200c were overexpressed in the serum of patients with adenomas compared to all the others [84].

Analysis of miRNAs in plasma of patients with adenomas was the task of the study conducted by Nagy et al. The authors identified three miRNAs (miR-31, -4506 and -452) differentially expressed in adenomas when compared with adjacent mucosa and the similar trend was also noticed in their plasma samples [85]. Four other miRNAs (miR-21, -29a, -92a and -135b) displayed significantly higher expression levels in adenomas when compared with non-affected adjacent mucosa. Alongside, patients with adenomas also evinced higher expression levels of miR-21 and -29a in their serum and exosomes than healthy individuals [86].

Besides plasma and serum, miRNAs can be also detected in the stool specimens [87]. In the study of Wu et al., authors identified miR-31 and -135b to be the most upregulated miRNAs in both CRC tissue and advanced adenomas tissue. These miRNAs were validated in stool specimens. The expression level of stool miR-135b was significantly higher in subjects with CRC compared with control individuals. However, there was no significant difference in the stool levels of miR-31. Authors repeated this analysis in patients upon removal of colorectal tumors and advanced adenomas and observed significant drop in miR-135b expression level in comparison with their level before removal [88].

### 3.3. Circulating Cell-Free DNA

As mentioned before, multiple genetic aberrations gradually accumulate over time, first in normal cells that develop into precursor lesions with the potential to develop into cancer. Thus, it may theoretically be possible to detect these genetic changes in plasma DNA taken from individuals with precursor lesions and monitor them over time to detect the progression. However, there are very few published studies on this issue.

One attractive way to improve adenoma detection and compliance is an analysis using cell-free DNA (cfDNA). Dying cells release their fragmented DNA into the circulation. In cancer patients, the cfDNA fraction that originates from tumor cells (circulating tumor DNA (ctDNA)) carries tumor-related alterations that can be detected using next-generation sequencing and PCR-based methodologies [102]. The cfDNA analysis, known as the liquid biopsy approach, is cost-effective, minimally invasive, and its specificity can be increased by tailoring the assay to detect tumor-specific mutations [51,103,104]. Recently, liquid biopsies have been used to detect minimal residual disease and monitor relapse after surgical resection of a localized disease [105]. The use of liquid biopsies for the detection of benign tumors has proved to be challenging [106,107]. The probability of detecting cfDNA is low in early-stage CRC [107] and many groups showed different results in terms of diagnostic value of total ctDNA levels or analysis of *KRAS* mutations in the plasma of patients with adenomas [105,108,109,110,111]. Encouraging studies reported an increase in total cfDNA or even detected tumor-related mutations in patients with benign adenomas [108,110].

Several parameters can influence the detection rate of benign lesions. Adenomas are typically small and do not manifest the persisted apoptosis or necrosis that is usually observed in advanced cancers. However, heterogeneity has been described in adenomas, which might affect *KRAS* mutation detection [112,113] suggesting that this oncogene might be subclonal and therefore inadequate for targeted cfDNA testing. Similar conclusions have been recently emerged from the study by Myint et al. [105] as authors argued that benign lesions do not release significant quantities of DNA in the circulation and are therefore unlikely to be diagnosed by liquid biopsies, at least using current technologies.

Further studies have shown even lower sensitivity. An analysis of 96 mutations in nine cancer driver genes (*BRAF, CTNNB1, EGFR, FOXL2, GNAS, KRAS, NRAS, PIK3CA*, and *TP53*) detected mutations in plasma cfDNA in 6% (12/200) of individuals undergoing colonoscopy; 42% of these individuals had polyps, and the rest had negative finding on colonoscopy [114]. *KRAS* mutations were detected in 33% (9/27) of individuals with CRC, 10% (3/30) of individuals with neoplastic polyps, and in 6% (2/35) of healthy individuals with no identified polyps during a colonoscopy. The same study also analyzed *BRAF* mutations in plasma cfDNA, and the results were similar in all three groups: 15% in those with CRC, 20% in individuals with neoplastic polyps, and 11% in healthy controls suggesting technical or biological issues. From a biological point of view, benign diseases, especially those with inflammatory background, may be associated with elevated levels of cfDNA [115]. In addition, somatic DNA mutations associated with cancer have been identified in histologically normal skin and colonic mucosa [116,117]. *KRAS* and *APC* mutations have also been identified in aberrant crypt foci in the colon which may be precursors of adenomas and CRC [118]. It further emphasizes that apparently unaffected colon mucosa may harbor cancer gene mutations and indeed *KRAS* mutations have been found in colonic effluent samples of patients at increased risk of CRC, however with normal finding on colonoscopy [119]. It was assumed that the source of the ctDNA could be from a neoplasm outside of the colorectal area, from apoptotic cells or destruction of precancerous cells, benign inflammatory lesions such as endometriosis, and small neoplasms with somatic DNA mutations during the normal process of immune surveillance [119,120]. 

In addition to *KRAS* and *BRAF* mutations, Galanopoulos et al. [109] recently studied blood samples and colonic biopsy specimens from healthy individuals with no polyps undergoing screening colonoscopy, patients with CRC, and patients with neoplastic intestinal polyps. Based on the mutation analysis for codon 12 of the *KRAS*, authors were able to discriminate patients with CRC compared to healthy individuals. However, with no success in predicting the presence of colonic polyps.

Kopreski et al. [110] found *KRAS* mutations in plasma cfDNA in 22 of the 62 patients with adenomas and in 9 out of 65 of those with hyperplastic or other non-neoplastic lesions. In prospective colonoscopy study, Perrone et al. [111] found 22 instances of high-grade intraepithelial neoplasia in adenomas (12.9%), 54 adenomas (31.8%), and 19 hyperplastic lesions (11.2%) in the 170 investigated individuals. *KRAS* mutations were found in the plasma of 3/19 patients with high-grade intraepithelial neoplasia (15.8%), 1/54 patients with adenomas (1.8%), and none of the patients with hyperplasia.

Gocke et al. [121] demonstrated that either of two hotspot mutations (codons 175 and 248) in *TP53* was detectable in cfDNA in 1.3% (3/240) of the individuals. However, only one of these three individuals had a polyp that carried the same *TP53* mutation, and thus, the origin of the other two plasma *TP53* mutations could not be determined. 

Mead et al. [108] analyzed diagnostic markers utilizing cfDNA isolated from samples obtained from 35 individuals without endoscopic abnormality, a group of 26 individuals with benign colorectal adenomas, and 24 patients with CRC. The best model to discriminate physiological from neoplasia populations was based on four DNA markers (Line1 79 bp, Alu 247 bp, mitochondrial DNA, and Alu 115 bp), with ROC curve of 0.810. The final test had a positive predictive value (PPV) of 81.1% for polyps and a negative predictive value (NPV) of 73.5% (sensitivity 83% and specificity 72%) for early cancer diagnosis. 

CRC screening with a multitarget stool DNA test was approved by the Food and Drug Administration in 2014. This simple, noninvasive, multitarget stool DNA (mt-sDNA)-based screening test (Cologuard; Exact Sciences, Madison, WI) has much greater sensitivity for the detection of both CRC and advanced precancerous lesions than FIT. Thus was developed to improve both non in-vasive screening performance and screening compliance [122,123]. Screening study data have similarly supported a Cologuard multiyear interval with a negative predictive value of a single test even of 99.94% for CRC and 95% for advanced adenoma [124]. Colo-guard consists of quantitative molecular assays to detect aberrantly methylated DNA (*NDRG4* and *BMP3*) and DNA mutations (*KRAS*) in stool plus a fecal hemoglobin immunoassay. Berger et al. suggested [122] that screening every 3 years using a multitarget mt-sDNA test provides reasonable performance at an acceptable cost.

Taken together, these results suggest that the detection of pathogenic mutations in plasma is not synonymous with precancerous lesions or cancer. Whilst many precancerous colorectal lesions are not detectable at all, some small polyps can shed detectable amounts of ctDNA in plasma. Since adenomas are potentially premalignant and should be excised, their detection through measurement of ctDNA should be useful and the finding of a positive test might increase the rate of screening colonoscopies, which suffers from poor patient compliance.

### 3.4. Circulating Tumor Cells (CTCs)

The process of tumor metastasis involves the release of epithelial cancer cells, called circulating tumor cells (CTCs) into the bloodstream. It has been observed that CTCs can access the circulatory system not only in metastatic stages but even at preinvasive lesions [125]. However, even in metastatic stages, the blood concentration of CTCs is extremely low and therefore the CTCs detection is difficult and much more challenging for colorectal adenomas or carcinomas in situ. Nevertheless, in terms of diagnostic value, CTCs provide an opportunity to monitor the development of cancer at all stages with a deeper understanding of tumor biology and better treatment efficiency [51].

The analysis of CTCs in the sense of liquid biopsy requires cell enrichment and CTC detection. The gold standard is represented by the CellSearch system [126], the only FDA approved method for CTC-detection today is based on immunomagnetic CTC enrichment using an antibody against the epithelial cell adhesion molecule (EpCAM) and combined with flow cytometry. EpCAM adhesion molecule is specific for epithelial cells and most carcinomas are characterized by its overexpression. Besides CTC detection by EpCAM, different assays based on physical characteristics (e.g., size, density, deformability, and electrical charge) or on more specific biological properties such as certain tumor epithelial protein (e.g., CK20, CD45) also exist [127].

In CRC, CTCs may originate not only from epithelial tumor cells, but also from tumor cells undergoing epithelial-mesenchymal transition, and tumor stem cells [128]. As mentioned before, extremely low concentration of CTCs in peripheral blood (e.g., 1-5 CTCs per 7.5 mL blood at CRC stage III) is the main obstacle in cancer progress investigation [129]. However, the presence of CTCs could serve as an indicator of metastatic spread of the disease. In a recent meta-analysis by Tan and Wu [130] that included 15 studies with 3129 CRC patients, the association between CTCs detection and poor survival outcomes for patients with CRC was proved. Another meta-analysis by Huang et al. [131], included 13 studies with eligible 2388 CRC patients observed CTCs level in peripheral blood before initiating chemotherapy and during the chemotherapy. They confirmed CTCs high level was significantly associated with poor progression-free survival and poor overall survival. Moreover, CTCs are suitable for evaluation chemosensitivity and gene expression to evaluate the current mutational status of the tumor [132]. Interesting results were shown by Guadagni et al. [133], where CTCs from 47% of CRC patients exhibited high sensitivity to mitomycin when compared to recommended chemotherapeutic for CRC.

Considering the low occurrence of CTCs in the bloodstream during CRC metastatic process, the utilization of CTCs for a screening of CRC early stages or precancerous lesions represents a very ambitious aim. The recent study of Tsai et al. [134] has accepted this challenging topic. On the day of the colonoscopy, 8 ml of peripheral blood was collected from 667 Taiwanese subjects (in detail the study consisted of 235 healthy controls, 107 subjects with adenomatous polyps, and 325 patients with CRC across all stages I-IV). All individuals were tested for CTCs presence by using the CellMax Platform (EpCAM(+), CK20(+), CD45(−) epithelial cells). Results of the study showed high specificity 86% and sensitivity 79% for adenomatous lesion and for CRC across all stages specificity 82% and sensitivity 95%. While the CTCs presence in metastatic CRC is widely accepted, CTCs detection at adenomatous polyps or in precancerous stages appears to be rather difficult. Nevertheless, CTCs detection has the potential to serve as a diagnostic tool in CRC screening.

### 3.5. Circulating Inflammation Markers

Inflammation belongs between one of the colorectal neoplasia drivers; however, particular inflammatory processes that play a role in early carcinogenesis are still unknown. Recently, Huang et al. [135] compared serum levels of 78 inflammation markers between 171 pathologically confirmed colorectal adenoma cases and 344 controls within the frame of Prostate, Lung, Colorectal and Ovarian Cancer Screening Trial. Their results provided important new evidence implicating C-chemokine cysteine motif chemokine ligand 20 (CCL20)—and growth-related gene oncogene products (GRO) –related pathways in early CRC, and further supported a role for insulin. The CCL20/CCR6 system also appeared to play a role in organ-selective liver metastasis of CRC. A recent meta-analysis of 4 cohorts and 10 case-control studies found no associations between adenoma and 3 most studied circulating inflammation markers CRP, IL-6, and TNF-α [136]. Similarly, no significant associations were found for C-peptide, GM-CSF, interferon-α (IFN-α), IL-1β, IL-2, IL-4, IL-6, IL-7, IL-10, IL-12, IL-17A, MIP-1β, and vascular endothelial growth factor and adenoma [137,138,139]. Although associations were reported for IFN-α2, IL-7, IL-8, MCP-3, and SIL-4R in cross-sectional analyses [137,140] they have not been prospectively confirmed.

### 3.6. Telomere Length

Telomeres are terminal repeated sequences at the ends of chromosomes and their shortening is associated with aging. During the cell division are telomeres shortened due to the lack of enzyme telomerase that synthesizes the ends of linear nucleic acid. Nevertheless, this enzyme is active in germ cells, stem cells, and in most of the cancer cells. In the advanced stage of CRC epithelial-mesenchymal transition occurs and telomere lengthening is maintenance by the alternative lengthening of telomeres (ALT) [141,142,143]. 

The latest studies reported that both short and long telomeres have been involved in carcinogenesis. The relationship between the length of telomeres in colorectal adenomas and the risk of cancer development is still subject to discussion [144]. Results of comparing the telomere length between adenomas, tumors, and adjacent mucosa are inconsistent and vary among several studies. In the study by Peacock et al. [141], telomere length of colon tissue from 40 adenomas and 45 controls identified during colonoscopy was exanimated. The result suggested that long telomeres in non-affected colon tissue are related to increased risk of CRC. Bautista et al. [145] observed higher telomerase activity in adenomas compared to their adjacent control mucosa, however shorter telomere length in adenomas in comparison to control tissue. Similar results were also obtained in the other studies [146,147], where telomere shortening in adenomas compared to adjacent mucosa was detected. The largest analysis of telomere length was done in the study of Suraweera et al. [148] where relative telomere length (RLT) was measured in 90 adenomas and adjacent normal mucosa. Adenomas showed a shortening of telomeres by 79% and lengthening only in 7% of cases.

## 4. Conclusions

The development of colorectal cancer is a comprehensive process of genetic, epigenetic, and structural modifications from benign adenoma to invasive cancer. Early detection and complete endoscopic removal of adenomas in their early stages is the key to survival with almost zero chance for cancer development. Nowadays, the most used method for the investigation of the large bowel is a colonoscopy. Although it is a very sensitive and reliable method, it brings considerable difficulties as a dietary restriction, invasive examination, risk of omission of some adenomas, high price, and requires a well-trained examiner. Non-invasive approaches for early adenoma detection are still evolving. The future perspectives in this area are moving towards liquid biopsy as a potential minimally invasive tool for clinical use. CfDNA, CTCs, inflammatory markers or specific RNA transcripts, such as a miR-31, miR-135, lncRNAs, released from adenoma lesions into circulation are extensively studied and have been shown as promising candidate biomarkers for early CRC. Although the concentration of cfDNA in plasma is very low, it still can provide useful information about mutations in crucial genes as a *KRAS* or *BRAF* that are involved in carcinogenesis. The early appearance of *KRAS* or *BARF* mutations in circulation even in healthy individuals warrants further investigation as a potential prognostic marker. Recently acquired knowledge about new possible biomarkers can help to better understand colorectal cancer evaluation and design its future detection strategy.

## Figures and Tables

**Table 1:** Classification of non-neoplastic and neoplastic polyps and polyposis [18].

Non-Neoplastic		Neoplastic		
Sporadic	Hereditary	Sporadic		Hereditary
Hyperplastic polyps	Hyperplastic polyposis	Benigns adenomas:	Tubular	Familial adenomatous polyposis (FAP)
			Villous	
			Tubulovillous	
Inflammatory polyps	Juvenile polyposis	Serrated adenomas:	Sessile serrated	Hereditary non-polyposis colorectal cancer (HNPCC)
Juvenile polyps			Traditional serrated	
	Peutz-Jeghers syndrome	Malignant lesions:	Carcinoma in situ	
				MUTYH associated polyposis
Lymphoid polyps			Intramucosal CRC	
			Invasive CRC	

**Table 2:** Current surveillance recommendation [20,30].

Neoplasia Found	Recommanded Interval for Colonoscopy Examination	Comment
Small rectal hyperplastic polyps	10 years	Exception are patients with hyperplastic polyposis syndrome, who need more intensive follow up.
One or two small (<1 cm) tubular adenomas with only low-grade dysplasia	5–10 years	The precise timing within this interval should be based on other clinical factors (such as prior colonoscopy findings, family history, and the preferences of the patient and judgment of the physician).
3 to 10 adenomas, or any adenoma ≥ 1 cm, or any adenoma with villous features, or high-grade dysplasia	3 years	Adenomas must have been completely removed. If the follow up colonoscopy is normal or shows only 1 or 2 small, tubular adenomas with low-grade dysplasia, then the interval for the subsequent examination should be 5 years.
More than 10 adenomas at one examination	< 3 years	The interval should be based on the clinician judgement and consider the possibility of an underlying familial syndrome.
Sessile adenomas that are removed piecemeal	2 to 6 months	Once complete removal has been established, subsequent surveillance needs to be individualized based on the endoscopist’s judgment. Completeness of removal should be based on both endoscopic and pathologic assessments.

**Table 3:** Summary of studies focusing on miRNA profiles in colorectal cancer (CRC) adenomas (in chronological order).

Reference	Origin of Study	Source	Number of Patients	miRNAs	Significant Relevant
[89]	USA	tissue	84 adenomas	miR-21	↑ expression associated with poor survival
[90]	Netherlands	tissue	25 CRC 30 adenomas	miR-17-92 cluster	↑ expression across adenoma carcinoma sequence
[81]	USA	tissue	222 CRC 41 adenomas 52 controls	miR-135b miR-31 miR-1 miR-137 miR-9 miR-99a	ability to distinguish adenomas vs. controls
[91]	USA	plasma	20 CRC 9 adenomas 12 controls	miR-532 miR-331 miR-195 miR-17 miR-142 miR-15b miR-532 miR-652 miR-15b miR-21 miR-339	ability to distinguish adenomas vs. controls and CRC
[83]	China	tissue	227 adenomas 37 controls	miR-194a	predictor for adenoma recurrence
[88]	China	tissue	40 CRC 16 adenomas	miR-31 miR-135b	↑ expression in adenoma carcinoma sequence
		stool	104 CRC 169 adenomas 109 controls		↑ expression in stool miR-135 level across the adenoma carcinoma sequence
[82]	Japan	tissue	870 CRC 637 adenomas	miR-31	↑ miR-31 expression was associated with CIMP status
[92]	USA	tissue	113 adenomas	miR-320a	↑ expression in adenoma carcinoma sequence
				miR-145 miR-192	↓ expression across adenoma carcinoma sequence
[93]	China	serum	307 CRC 164 adenomas 266 controls	miR19a miR-92a miR-223a	↑ expression in adenoma carcinoma sequence
				miR-422	↓ expression across adenoma carcinoma sequence
[80]	UK	tissue	13 CRC 55 adenomas 10 controls	miR-135b	↑ expression across adenoma carcinoma sequence
[94]	Netherlands	tissue	52 CRC 48 adenomas	miR-15a	↑ expression in adenoma carcinoma sequence
[95]	France	tissue	41 CRC 51 adenomas 34 controls	miR-15b miR-16b miR-21 miR-24 miR-145 miR-150 miR-378	↓ expression in adenomas compared to controls
[96]	Japan	tissue	151 CRC 21 adenomas	miR-148a	↓ expression across adenoma carcinoma sequence
[97]	USA	serum	11 CRC 20 adenomas 10 controls	miR-30b miR-30c miR-146a miR-30d	↑ expression in adenoma carcinoma sequence
[98]	Japan	tissue	18 CRC with adenomas 3 CRC without adenomas 21 normal mucosa	miR-320 family	↓ expression in adenomas and early CRC tissue vs. controls
[78]	USA	tissue	109 adenomas	miR-145 miR-143 miR-107a miR-194 miR-26a miR-663b miR-1268 miR-320a miR-1275	ability to distinguish high risk adenomas from low risk adenomas
[86]	Japan	serum (+exosomes)	26 adenomas 47 controls	miR-21 miR-29a miR-92a miR-135b	ability to distinguish adenomas vs. controls discriminate patients with ↑ risk adenoma
[85]	Hungary	tissueplasma	20 CRC 20 adenomas 20 controls	miR-31 miR-10b miR-183 miR-196a	expression of miRNAs in plasma correlated with matched tissue expression level ability to distinguish adenomas vs. controls and CRC
[99]	China	serum	20 CRC 20 adenomas 20 controls	miR-4463 miR-5704 miR-371b miR-1247 miR-1293 miR-548a miR-107 miR-139	ability to distinguish CRC vs. adenomas vs. controls
[100]	USA	serum	34 CRC 33 adenomas 35 controls	Ratios of: let-7b/miR-367 miR-130a/miR-409 miR-148/miR-27 miR-148/miR-409 miR-21-miR367	ability to distinguish adenoma vs. controls
				miR-17/miR-135b miR-92a/miR-135b miR-451a/miR-491	ability to distinguish CRC vs. adenomas
[84]	Colombia	tissue serum	45 CRC 25 adenomas 45 controls	miR-141 miR-200c	↑ expression in adenomas compared to CRC and controls
[79]	USA	tissue	26 adenomas 30 controls	miR-31 miR-135b miR-378a	predictors of serrated neoplasia
[101]	Ireland	plasma	16 CRC 24 adenomas 8 controls	miR-34 miR-150	ability to distinguish CRC vs. adenomas
[76]	China	tissue	6 CRC 6 adenomas 6 controls	miR-135b miR-18a miR-29b	↑ expression in adenoma carcinoma sequence
				miR-1 miR-338 miR-218	↓ expression level across adenoma carcinoma sequence

↑ high/higher, ↓ low/lower.

## Abbreviations

ALT	alternative lengthening of telomeres
aFAP	attenuated familial adenomatous polyposis
CC	colon cancer
CCE	colon capsule endoscopy
CCL20	C-chemokine cysteine motif chemokine ligand 20
cfDNA	cell-free DNA
CGH	comparative genomic hybridization
CIMP	CpG island methylator phenotype
CIN	chromosomal instability
CRC	colorectal cancer
CTCs	circulating tumor cells
CT	computed tomographic
ctDNA	circulating tumor DNA
EpCAM	epithelial cell adhesion molecule
FAP	familial adenomatous polyposis
FDA	Food and Drug Administration
FFPE	formalin-fixed paraffin-embedded
FIT	fecal immunochemical test
FOBT	fecal occult blood test
gFOBT	guaiac fecal occult blood test
Hb	hemoglobin
HNPCC	hereditary non-polyposis colorectal cancer
HPs	hyperplastic polyps
JPS	Juvenile polyposis syndrome
LOH	loss of heterozygosity
LS	Lynch syndrome
MAP	MUTYH-associated polyposis
MMR	mismatch repair pathway
mt-sDNA	multitarget stool DNA
ncRNA	non-coding RNA
NER	nucleotide excision repair genes
NGS	next-generation sequencing
NPV	negative predictive value
PHTS	hamartoma tumors syndrome
PJS	Peutz-Jeghers syndrome
PPAP	Polymerase proofreading-associated polyposis
PPV	positive predictive value
RC	rectal cancer
RLT	relative telomere length
SSA/Ps	sessile serrated polyps
UK	United Kingdom
USA	United States of America
WNT	Wingless/Int-1 pathway
